# surviBALL: exploring lncRNA expression at diagnosis for 5-year EFS risk stratification in pediatric B-ALL—a proof of concept

**DOI:** 10.1186/s40348-025-00210-3

**Published:** 2025-11-10

**Authors:** Unai Illarregi, Nerea Bilbao-Aldaiturriaga, Angela Gutierrez-Camino, Ivan Martinez de Estibariz, Javier Arzuaga-Mendez, Mireia Camos, Manuel Ramirez-Orellana, Itziar Astigarraga, Chantal Richer, Daniel Sinnett, Idoia Martin-Guerrero, Elixabet Lopez-Lopez

**Affiliations:** 1 Department of Genetics, Physical Anthropology and Animal Physiology, Faculty of Science and Technology, University of the Basque Country (UPV/EHU), Leioa, Basque Country, Spain; 2 Pediatric Oncology Group, Biobizkaia Health Research Institute, Barakaldo, Basque Country, Spain; 3 Hematologic Neoplasm Group, Biobizkaia Health Research Institute, Barakaldo, Basque Country, Spain; 4 Hematology Laboratory, Sant Joan de Déu Research Institute, Esplugues de Llobregat, Barcelona, Spain; 5 Department of Pediatric Hematology and Oncology, Niño Jesús University Hospital, Madrid, Madrid, Spain; 6 Department of Pediatrics, Pediatric Hemato-Oncology Unit, Hospital Universitario Cruces, Biobizkaia Health Research Institute, Barakaldo, Basque Country, Spain; 7 Department of Pediatrics, Faculty of Medicine and Nursing, University of the Basque Country (UPV/EHU), Leioa, Basque Country, Spain; 8 Division of Hematology-Oncology, CHU Sainte-Justine Research Center, Montreal, QC H3T 1C5 Canada; 9 Department of Pediatrics, Faculty of Medicine, University of Montreal, Montreal, QC H3C 3J7 Canada; 10 Department of Biochemistry and Molecular Biology, Faculty of Science and Technology, University of the Basque Country (UPV/EHU), Leioa, Basque Country, Spain

**Keywords:** B-cell acute lymphoblastic leukemia, Long non-coding RNA, Event-free survival, Pediatric oncology, Prognostic biomarker

## Abstract

**Background:**

B-cell Acute Lymphoblastic Leukemia (B-ALL) remains an important cause of cancer-related death in children. Therefore, accurate identification at diagnosis of patients at high risk of relapse is crucial. In this context, long non-coding RNAs (lncRNAs) could be novel candidates with great potential. Hence, the aim of this study was to identify new prognostic biomarkers in pediatric B-ALL through an RNA sequencing (RNA-seq) approach that allows the detailed exploration of a wide range of lncRNAs.

**Methods:**

Total RNA from two cohorts of B-ALL patients (C1 with 50 Spanish patients, and C2 with 72 Canadian patients) was sequenced with a depth of approximately 150 million paired-reads using Illumina technology. All protein coding and non-coding genes included in lncRNAKB annotation were studied to develop a gene expression-based 5-year Event Free Survival (EFS) prediction model.

**Results:**

First, univariate Cox proportional hazards analyses identified 48 genes significantly associated with higher EFS risk in both cohorts. From these, ALASSO regression selected five genes, all of which are lncRNAs, as the most informative to develop the prediction model, which we have called surviBALL. Stratification of patients into three risk groups according to the surviBALL model revealed significantly poorer EFS in high-risk patients across C1, C2, and the integrated C1 + C2 cohort (*P* < 0.001). Validation in an independent cohort of 177 publicly available B-ALL samples confirmed surviBALL’s prediction capacity (*P* = 2.80 × 10^− 4^) and its independence of both subtype and MRD.

**Conclusions:**

These findings suggest that surviBALL has the potential to complement current risk stratification approaches, particularly by identifying patients at high risk of relapse at diagnosis. As a hypothesis-generating proof of concept, this study highlights the promise of more personalized treatment strategies and warrants further validation in independent cohorts.

**Supplementary Information:**

The online version contains supplementary material available at 10.1186/s40348-025-00210-3.

## Background

Childhood Acute Lymphoblastic Leukemia (ALL) is the most common pediatric malignancy, accounting for 25% of all childhood cancers. It is a highly heterogeneous hematological malignancy, arising from the transformation of either B-cell (B-ALL) or T-cell (T-ALL) precursors, B-ALL being the most frequent (> 85% of cases) [1, 2].

Over the past decades, survival rates have improved from 10% to over 90%, in part due to patients’ stratification into risk groups for therapy optimization, which maximizes cure rates while minimizing toxicities [3, 4]. Current ALL treatment protocols consider factors such as age, white blood cell count, karyotype, specific rearrangements, or minimal residual disease for risk stratification. In addition, in recent years, more than 20 genetically distinct B-ALL subtypes with prognostic significance have been identified [3, 5], based on gene expression profiling at diagnosis. Nevertheless, ALL remains the leading cause of cancer-related death in children [6], and some patients still experience relapse, even after being classified in low-risk groups at diagnosis. Therefore, there is a need for improvement in risk group stratification.

Noteworthy, gene expression-based subtypes are mainly based on protein coding (PC) genes, and non-coding RNAs (ncRNAs) have recently emerged as promising candidates for biomarker discovery, given their abundance and diverse functional roles [7]. While many types of ncRNAs have been identified, microRNAs (miRNAs) remain the most extensively studied. However, long-non-coding RNAs (lncRNAs) are gaining relevance due to their ability to modulate gene expression through diverse mechanisms [8]. Consequently, lncRNAs may have the potential to influence pathways of prognostic relevance in ALL and have been suggested as candidate prognosis markers in other pediatric leukemias [9]. Despite their potential, few studies have been conducted to explore their role as prognostic biomarkers in ALL, highlighting the need for further research [10].

Here, we investigated the transcriptional landscape of two independent pediatric B-ALL cohorts to define candidate lncRNAs for risk stratification at diagnosis.

## Methods

### Population of study

The study analyzed two retrospective case series of patients diagnosed with B-ALL. Cohort 1 (C1) included 50 patients diagnosed in three Spanish hospitals: 42 were consecutively diagnosed at Cruces University Hospital between September 2013 and March 2022 (other 14 cases were excluded due to insufficient diagnostic material or poor RNA quality, none of them were refractory or experienced early events). The remaining 8 patients were selected from Niño Jesús Children’s University Hospital and Sant Joan de Déu Children’s Hospital, to increase the number of patients in underrepresented molecular subtypes, thereby contributing to a more balanced and representative study population. All patients were treated according to the SEHOP-PETHEMA 2013 (patients aged 1 to 16) or Interfant06 (patients under 1 year of age) protocols. Cohort 2 (C2) included 72 patients diagnosed from 2001 to 2017 in the CHU Sainte-Justine in Canada, for which RNA-seq was performed at diagnosis with Illumina technology, and treated according to Dana-Farber Cancer Institute (DFCI) ALL Consortium Protocols (DFCI-00-01, DFCI-05-001 or DFCI-11-001, depending on the date of diagnosis). Demographic and clinical data were obtained from patient’s medical files by clinicians; collected data included sex, age, treatment protocol, protocol-based risk group, indicators of long-term response (5-year event-free survival (EFS), and 5-year overall survival (OS)), and remission indicators for C1 cases. All data were complete, except for one case with missing blasts% value at day + 15, and another case with missing negative MRD date after a positive result at day + 33 (Table 1; Additional File 1.a). Additionally, 177 publicly available B-ALL samples from Therapeutically Applicable Research to Generate Effective Treatments (TARGET) “ALL: Expansion Phase II” project, treated according to protocols of Children’s Oncology Group (COG), were analyzed to evaluate the consistency of the findings in an independent validation cohort [11]. Survival and clinical data was retrieved from the GDC data portal, and raw fastq files downloaded from SRA, under dbGaP accession number phs000218 (Additional File 1.b). Subtype for all the patients was assigned using the classifier published by Tran et al., 2022 [12] and Arriba [13] to detect fusion genes and divide patients in fusion-like subtypes.

**Table 1:** Clinical information of the patients included in the study

	Cohort 1 (C1; *n* = 50)	Cohort 2 (C2; *n* = 72)
Sex		
Female	22	36
Male	28	36
Age at diagnosis		
< 1	2	1
1–10	34	47
> 10	14	24
Follow-up (5 years)		
Event	7	12
*Exitus*	4	4
Median follow-up	60 months (67*)	60 months
Protocol-based risk group		
Very high	-	5
High	7	32
Intermediate	41	-
Standard	2	35
Protocol		
SEHOP-PETHEMA 2013	48	-
Interfant06	2	-
DFCI-00–01	-	21
DFCI-05-001	-	25
DFCI-11-001	-	26

*w/o administrative censoring

### RNA extraction and sequencing

RNA was extracted from patients’ bone marrow or blood samples at diagnosis (> 50% blast cells) using TRIzol™ Reagent (Thermo Fisher Scientific, Carlsbad, CA, USA) or QIAamp RNA Blood Mini Kit (QIAGEN, Aarhus, Denmark) in C1, and mini AllPrep RNA kit (QIAGEN) in C2. The integrity and quality of the isolated RNA was determined using the Bioanalyzer 2100 kit (Agilent Technologies, Santa Clara, CA, USA). Only RNAs with RIN > 7 were selected for library preparation. RNA libraries (TruSeq Stranded Total RNA Library Prep Kit, Illumina, San Diego, CA, USA) were prepared using the Ribo-Zero Gold Kit (Illumina) according to the manufacturer’s protocol. The resulting libraries (stranded and ribosomal RNA-depleted) were sequenced at approximately 150 M reads per sample (paired-end 2 × 75 bp or 2 × 100 bp) on NovaSeq 6000 system (Illumina) at the Integrated Center for Clinical Pediatric Genomics at CHU Sainte-Justine.

### RNA sequencing bioinformatic analysis pipeline

Standard bioinformatic tools were used for data analysis. Briefly, alignment to the hg38 human reference genome was performed using Spliced Transcripts Alignment to a Reference (STAR v2.7.9a) [14]. Subsequent analyses were performed using R software (R version 4.1.3) [15]. Expression of all the genes in lncRNA Knowledgebase (lncRNAKB) annotation, including PC genes and a large number of lncRNAs in hg38 human reference genome coordinates [16], were quantified with featureCounts [17]. Finally, genes expressed in at least three samples in both C1 and C2 were selected, and batch effects that may be caused due to different sequencing times or read-length were corrected with ComBat-seq [18]. Size factor scaling was applied using DESeq2 to obtain normalized counts for downstream analyses [19].

### Identification of EFS-associated genes

Univariate Cox proportional hazards models (UVC) were fitted iteratively for each minimally expressed gene in C1 and C2 using normalized expression as continuous variable, and right censoring follow-up data at five years from diagnosis. The cases that did not reach 5 years follow-up were included in the analysis being censored at their last known follow-up time. After analyzing each cohort independently, genes significantly associated (*P* < 0.01 & Hazard-ratio (HR) < 1/>1) with 5-year EFS in both cohorts with the same HR trend were selected as candidates for further analyses.

### ALASSO-based gene selection and model development

Previously selected candidate genes were used as input variables in the Adaptive Least Absolute Shrinkage and Selection Operator (ALASSO) regression on C1 to reduce the number of predictors in the final model, while keeping predictive ability [20]. Ridge regression with cross-validation was performed to determine an optimal lambda value, which was used as the penalty factor in the ALASSO procedure. Then, ridge coefficients corresponding to this lambda value were extracted, and the ALASSO regression with 10-fold cross-validation was conducted. Genes selected in the ALASSO regression were used as input variables to develop the EFS-predictor multivariate Cox proportional hazards model in C1. Administrative right censoring was applied to all the samples at year five from diagnosis if follow-up was longer, and proportionality assumption was checked with the *cox.zph()* function [21]. TRIPOD + AI guidelines were followed and the checklist is included as Additional File 2.

### Performance assessment

Discriminative ability was assessed with 5-year time-dependent Receiver Operating Characteristic (ROC) curve’s Area Under the Curve (AUC) value and Uno’s Concordance index. For calibration assessment at a fixed time point (five years) the Integrated Calibration Index (ICI) was calculated, which measures the mean absolute difference between smoothed observed proportions and predicted probabilities, also the E50 and E90, which denote the median and the 90th percentile of the absolute difference between, observed and predicted probabilities of the outcome at time *t*. Finally, the Brier Score and the Index of Prediction Accuracy (IPA) or scaled Brier score were calculated for overall performance assessment, for which internal validation was conducted using the bootstrap resampling approach. The difference between the apparent performance and the internal validation performance reflects the optimism in the original model’s predictions. All these metrics and formulas were used as described in McLernon et al., 2023 [22].

### Risk score, samples stratification and survival curves

The coefficients from the model developed in cohort C1 were used to calculate the predicted event-risk values for each patient in C1, C2, the integrated C1 + C2, and TARGET cohorts with the following formula:

$$\:Risk\:score={\sum\:}_{i=1}^{n}{normExpr}_{i}\text{*}{{\upbeta\:}}_{i}$$

Patients were ranked according to their predicted event-risk scores. The ones with the highest predicted risk scores, falling within the top decile, were categorized as “High risk”. Those identified in the 10–30% range of the highest predicted event-risk scores were categorized as “Intermediate risk”, while the remaining patients were classified as “Low risk”. Kaplan-Meier survival curves were plotted, and statistical significance was evaluated with the log-rank test, to compare these three risk groups in the individual and combined cohorts.

The prognostic value of clinical variables (age at diagnosis, sex, and subtype) was evaluated in UVC analyses individually and together with the predicted risk value and groups on the combined C1 + C2 cohort, as well as in the TARGET validation cohort, where MRD was also evaluated. To ensure model stability and avoid issues associated with sparse data, molecular subtypes with limited representation were grouped together with non-classified patients under a common category labeled “Others”.

### Function prediction for candidate lncRNAs

Guilt-by-association methodology was used for candidate lncRNAs function prediction, the principle of which is that if lncRNAs and coding genes have similar expression patterns, they have similar functions [23]. Integrated cohort (C1 + C2) was used to calculate correlations between each lncRNA and every PC gene minimally expressed. Genes correlated with absolute r-value > 0.5 and p-value < 0.01 were used as input for overrepresentation analysis on ConsensusPathDB web tool for each lncRNA [24]. Additionally, expression differences across subtypes were evaluated using the Kruskal-Wallis test followed by Dunn’s post-hoc test with Bonferroni correction.

## Results

### Identification of EFS-associated genes

94 073 and 75 886 minimally expressed genes were identified in C1 and C2, respectively, of which 901 in C1 and 1 700 in C2 were significantly associated with 5-year EFS. Among them, 48 genes significantly associated with EFS in both cohorts, exhibiting consistent effect trends. All the overlapping genes presented a Hazard-Ratio (HR) > 1, meaning that higher expression values were correlated to worse EFS, while no overlap was seen for genes with HR < 1 (Additional File 1.c).

### ALASSO-based gene selection, surviball model development and performance assesment

We employed an ALASSO variable selection method to identify the most relevant predictors associated with the 5-year EFS in C1 dataset starting from the 48 overlapping genes obtained in the EFS analysis. Five predictor variables were selected by the ALASSO regression, all of which were lncRNAs (Table 2). A Multivariate Cox proportional hazards model, hereafter referred to as surviBALL, was developed with those five genes in cohort C1, treating them as continuous variables and applying right censoring at year five.

**Table 2:** Candidate genes selected using ALASSO regression, with univariate Cox proportional hazards results in C1 and C2 for 5-year EFS

Gene ID	Other aliases	Chr.	Start	End	C1 HR	C1 *P*	C2 HR	C2 *P*
lnckb.8576	*CTD-2589M5.5 ENSG00000254639 AC116021.1*	chr11	46,238,382	46,239,267	1.07	0.0048	1.20	0.00075
lnckb.32044	*CATIP-AS1 ENSG00000225062*	chr2	218,366,665	218,367,902	1.11	0.00079	1.11	0.0033
lnckb.61426	*CATG00000027638.1*	chr16	67,393,673	67,394,034	1.32	0.0048	1.30	0.0028
lnckb.66409	*CATG00000110942.1*	chrX	39,755,952	39,756,532	1.24	0.0015	1.33	0.0066
lnckb.83321	*NONHSAG107984.1*	chr15	32,441,018	32,441,680	1.18	0.00014	1.41	0.004

*EFS* Event-free survival, *Chr *chromosome, *C1 *Cohort 1, *C2 *Cohort 2, *HR* Hazard-ratio, *P *p-value

surviBALL’s performance was assessed using a range of statistical metrics recommended by McLernon et al., 2022 for evaluation of predictive models with survival outcomes [22]. Among discrimination metrics, AUC and Concordance indexes (C-index) were calculated. The model exhibited excellent discriminative ability, as indicated by an AUC of 0.929 in the 5-year time-dependent Receiver Operating Characteristic (ROC) curve, and 0.932 (se = 0.041) value in Uno’s weighted C-index on C1. Regarding calibration, the Integrated Calibration Index (ICI) was 0.014 (95% CI: 0.011–0.019; E50: 0.009; E90: 0.026), further supporting surviBALL’s calibration performance. Finally, the Brier score was computed to assess surviBALL’s overall performance, obtaining a value of 0.032 (95% CI: 0–0.074), with a null model Brier score B_0_ of 0.105. The scaled Brier score or IPA, which allows easier interpretation, was 69.6% (95% CI: 54.5% − 88.5%), indicating a better performance compared to a null model. The internal bootstrap validation gave a 0.045 Brier score and an IPA of 56.9%, which still indicates strong predictive performance, though the 12.7% optimism reflects a slight overfitting.

### Patient stratification and risk assessment

**Fig. 1:**
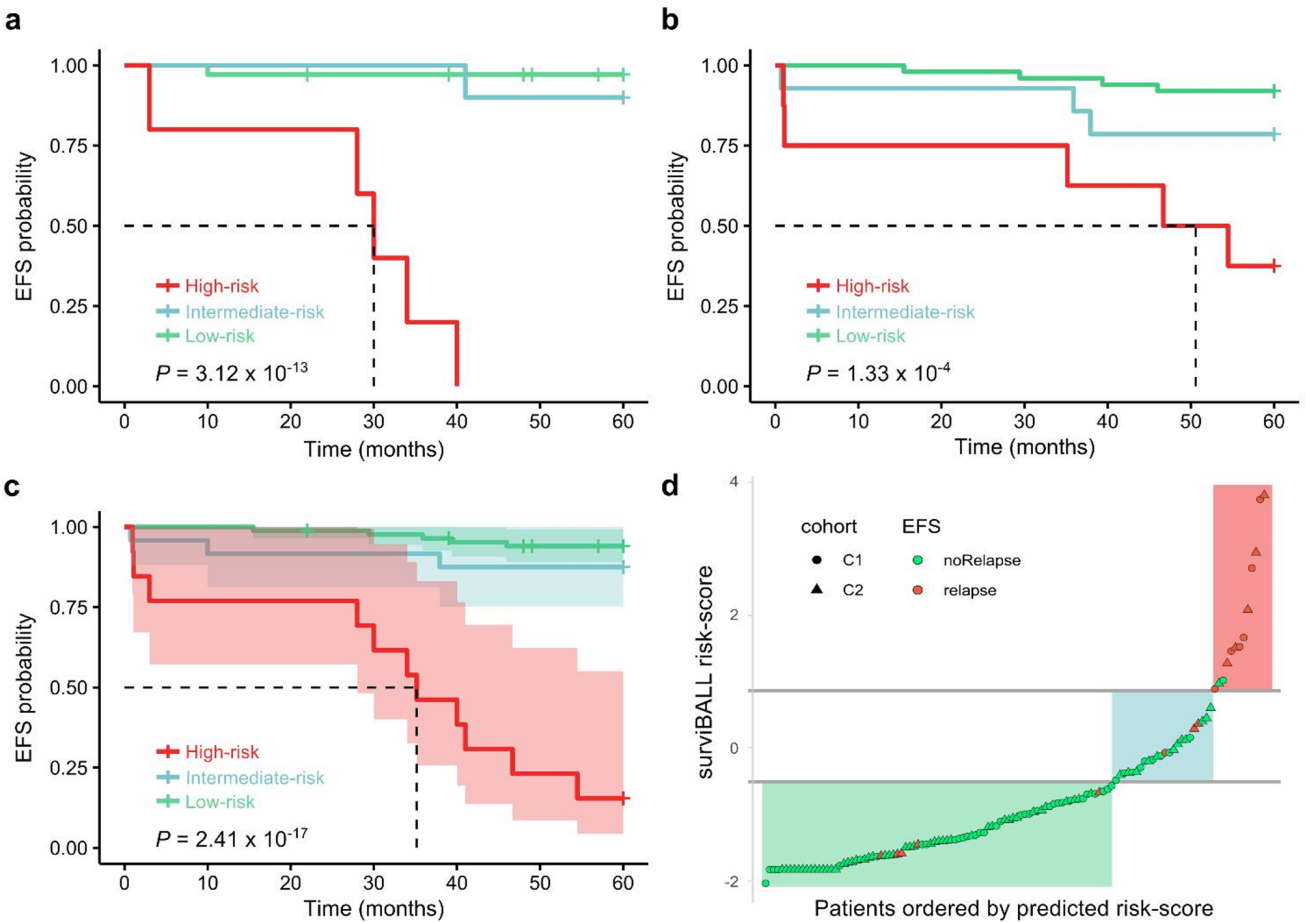
Kaplan-Meier curves comparing risk groups after surviBALL stratification. **a-c.** Kaplan-Meier survival curve for 5-year EFS in C1 **a**, C2 **b** and combination of both cohorts **c** comparing risk groups after surviBALL stratification. Shading represents the 95% CI, and dash lines the median survival times. **d.** Representation of predicted risk values as circles (C1) or triangles (C2) for each patient. Horizontal grey lines divide the risk groups, while red points indicate relapsed patients and green points indicate non-relapsed patients. Background shading highlights low- (light green), intermediate- (light blue), and high-risk (light red) regions. EFS: Event-free survival; C1: Cohort 1; C2: Cohort 2; CI: Confidence interval

After the development of surviBALL, predicted risk scores were calculated for C1, C2, and the combined C1 + C2 cohort. Subsequently, a stratification based on predicted risk scores was carried out for each group, resulting in the classification of samples into three distinct risk categories, “High-risk” (top decile), “Intermediate-risk” (10–30%) and “Low-risk”. The three patient groups presented statistically significant differences in EFS according to the Kaplan-Meier survival analysis, with global log-rank p-values of 3.12 × 10^–13^, 1.33 × 10^− 4^, and 2.41 × 10^–17^ in C1, C2 and C1 + C2 groups, respectively (Fig. 1). Patients in the High-risk group presented very poor EFS in comparison with the other two groups. No significant difference was observed for EFS between C1 and C2 (*P* = 0.745), or between different protocols in C2 (*P* = 0.560) (Additional File 3.a).

Individual analyses of clinical variables showed no significant relationship with 5-year EFS for age at diagnosis or sex. However, subtype was significantly associated with EFS (*P* = 0.02; C = 0.718). Integration of surviBALL predicted values with subtype in a multivariate Cox proportional hazards analysis, confirmed that surviBALL is independently associated with 5-year EFS (HR = 2.98, 95% CI: 2.006–4.420; *P* = 6.17 × 10^− 8^; Additional File 1.d).

**Fig. 2:**
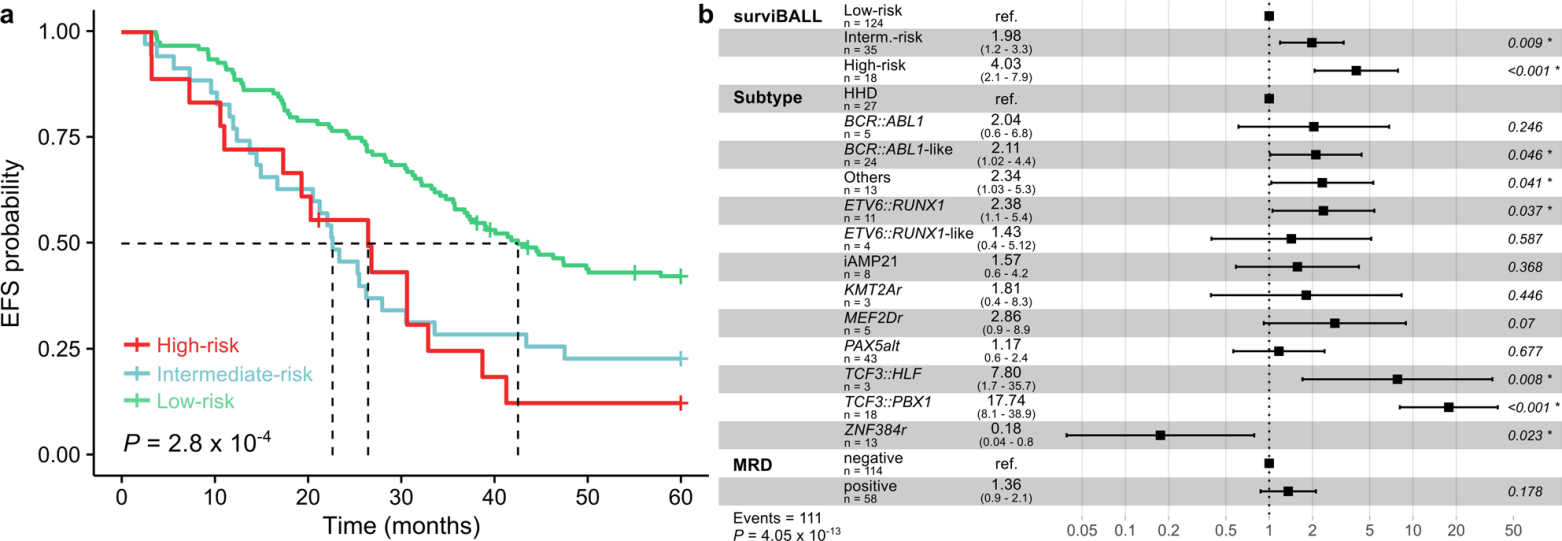
Survival analyses in the TARGET validation cohort. **a** Kaplan-Meier survival curve for 5-year EFS in TARGET cohort comparing risk groups after surviBALL stratification. **b** 5-year EFS Cox proportional hazards analysis with predicted risk groups, subtype, and MRD variables. EFS: Event-free survival; MRD: Minimal residual disease

Independent validation of surviBALL’s risk prediction ability was conducted using the TARGET B-ALL cohort. As expected, clinical outcomes in this cohort were poorer compared to the general population. However, stratification into three risk groups based on the predicted risk scores revealed a consistent trend in outcomes, supported by a significant global log-rank p-value in the Kaplan-Meier survival analysis (2.8 × 10^− 4^; Fig. 2a). Notably, 87.7% of the High-risk group patients experienced an event within the first five years from diagnosis, which is in concordance with what was observed in the combined C1 + C2 cohort.

Additionally, Cox proportional hazards models in the TARGET cohort validated the results obtained in the integrated C1 + C2 cohort. Age and sex were not significantly associated with EFS in univariate Cox analyses. In contrast, subtype and positive minimal residual disease (MRD; defined as > 0.01% at end of induction, day + 29) were significantly associated with 5-year EFS, and risk values and groups assigned by surviBALL predictions remained independently significant when analyzed alongside subtype and subtype + MRD in the TARGET cohort (Fig. 2b; Additional File 1.e).

### Gene biotype comparison

lncRNAKB annotation includes 95,776 genes classified in four different gene biotypes: PC (21.2%), lncRNA (75.3%), miscellaneous RNA (miscRNA; 1.3%), and antisense RNA (2.2%). Of 94 073 minimally expressed genes in C1, 75.39% were lncRNAs and 21.27% PC genes, whereas in C2 70.6% of the 75 886 minimally expressed genes were lncRNAs and PCs accounted for 25.54% of the genes expressed in at least three samples. Overlap after univariate Cox proportional hazards analyses between both cohorts comprised a higher proportion of lncRNAs: 89.6% of lncRNAs (43), 8.3% of PC genes (4), and 2.1% of miscRNAs (1). Finally, 100% of the ALASSO candidate genes were lncRNAs (Additional File 3.b).

### Function prediction for candidate lncRNAs

**Fig. 3:**
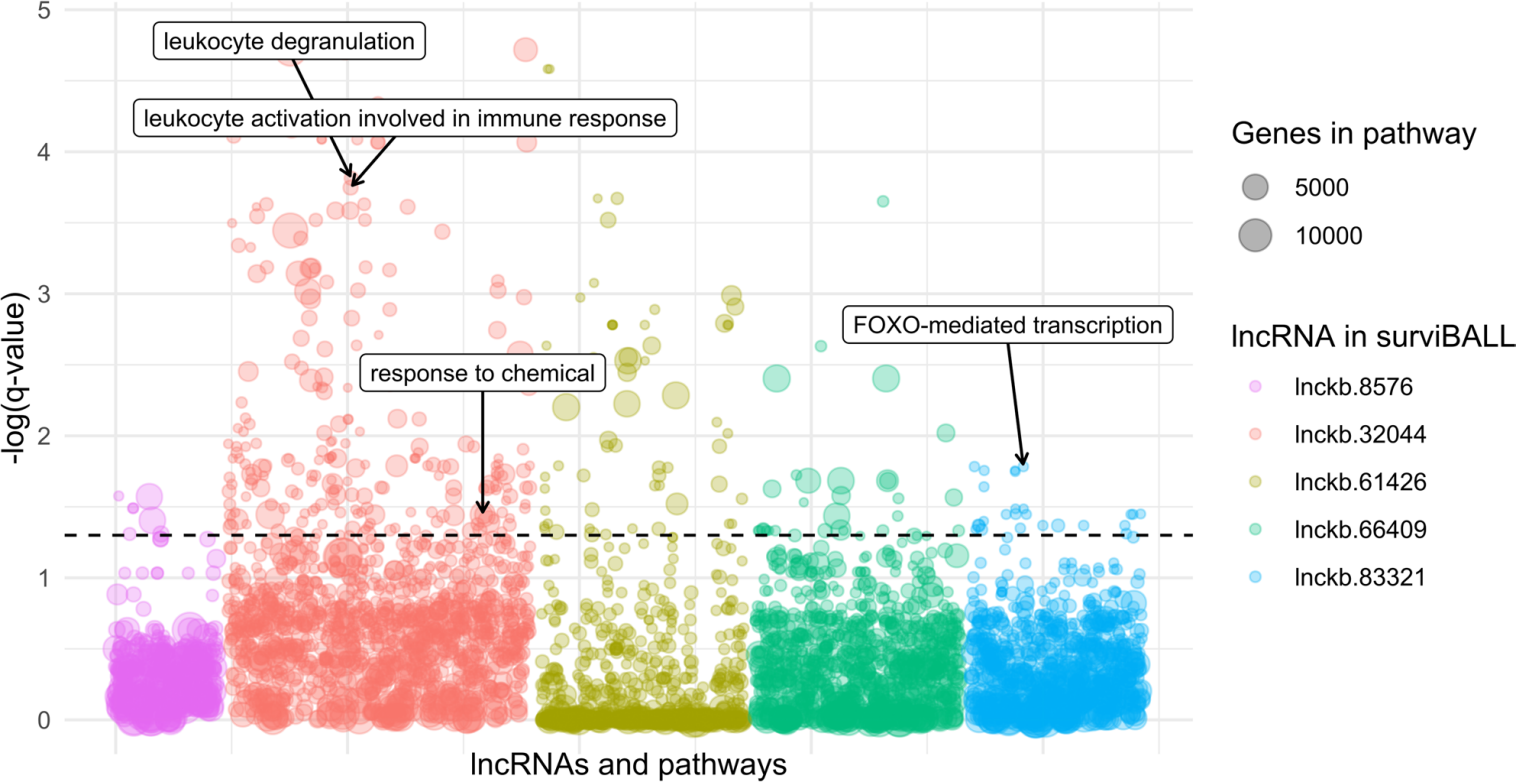
Bubbles plot showing overrepresentation analyses results for the lncRNAs in surviBALL after guilt-by-association correlation. Each bubble represents a pathway or Gene Ontology term, with relevant significant pathways highlighted

Guilt-by-association analysis reported 385 positive correlations between the five lncRNAs in surviBALL and minimally expressed PC genes, ranging from 26 to 165 per lncRNA (Additional File 1.f). The highest correlation value was 0.84, observed in two lncRNA-PC pairs: *lnckb.61426*-*DSPP* and *lnckb.66409*-*RPL18*. Relevant PC genes correlated to the lncRNAs from surviBALL included *CFD* (*lnckb.32044* and *lnckb.66409*), *NOTCH3* (*lnckb.61426*), and *PVR* (*lnckb.66409*). Interestingly, those genes’ overrepresentation analyses showed significant association to pathways and Gene Ontology functions as leukocyte activation involved in immune response, leukocyte degranulation, response to chemical, or FOXO-mediated transcription (Fig. 3; Additional File 1.g).

Comparison of gene expression across subtypes in the TARGET cohort revealed significant differences for two of the lncRNAs in surviBALL. *lnckb.8576* showed higher expression in *BCR::ABL1* patients compared to several subtypes (Additional File 3.c), while *lnckb.32044* expression was higher in iAMP21 cases compared to *TCF3::PBX1* patients (*P* = 0.003; Additional File 3.d). However, no significant differences were observed in the combined C1 + C2 cohort (Additional File 1.h).

## Discussion

In this study, we have developed and evaluated surviBALL, a Cox proportional hazards model based on the expression of five lncRNAs (*lnckb.8576*, *lnckb.32044*, *lnckb.61426*, *lnckb.66409*, *lnckb.83321*), which shows potential as a prognostic predictor of 5-year event-free survival in pediatric B-cell acute lymphoblastic leukemia. As a hypothesis-generating proof of concept, this model highlights the relevance of lncRNA expression at diagnosis and warrants further validation in independent cohorts.

surviBALL was developed using thorough gene expression data from two independent cohorts of consecutive B-ALL patients, treated with different treatment protocols. Stratification in three different risk groups using this model showed notable differences in outcome among risk groups. Interestingly, its prognostic value was independent of other variables, such as molecular subtype, which is already used as biomarker for risk stratification [4]. Unluckily, minimal residual disease was not analyzed in the C1 + C2 combined cohort due to the unavailability of these data for most of the patients, and its independence could not be confirmed. surviBALL was validated in B-ALL samples of the TARGET ALL Phase II, which is a relapse-enriched cohort. In this case, even if the average survival was poor in all risk groups, surviBALL high-risk patients presented a poorer outcome than the remaining patients did, and its predictive value was independent of both subtype and MRD. Therefore, surviBALL presented prognostic value in different cohorts of patients treated with different treatment protocols (SEHOP-PETHEMA, DFCI and COG group protocols). This suggests that surviBALL could serve as an additional risk assessment tool in clinical settings, although its utility should be confirmed in larger independent cohorts.

Interestingly, although our study design included both protein coding and lncRNA genes, each step of our analysis—beginning with minimal expression thresholds and candidate filtering through UVC and ALASSO regression—highlighted an increasing proportion of lncRNAs, leading to a model that includes only lncRNAs. This progression underscores their potential as robust prognostic biomarkers, which aligns with findings from other studies [9], even though the functional mechanisms of most of these lncRNAs remain unexplored.

Of note, the five lncRNAs comprising surviBALL have not been previously associated with pediatric B-ALL. However, *lnckb.8576* and *lnckb.32044* were reported as deregulated in other malignances, Acute Myeloid Leukemia and thyroid cancer, respectively [9, 25, 26], suggesting that these lncRNAs may play broader roles beyond pediatric B-ALL, making them candidates for further investigation. In addition, it is important to note that the sequencing and bioinformatics approach used here allowed for the analysis of a broader set of genes compared to other studies. This included the detection of non-poly-A lncRNAs using rRNA-depletion instead of poly-A selection, the analysis of a larger number of genes, enabled by the selection of lncRNAKB annotation [16], which integrates lncRNAs from six databases, and the ability to analyze very lowly expressed genes due to the high coverage obtained for each sample. This methodological advantage could have potentially enhanced the detection of lncRNA expression, which would align with a lack of detection of these lncRNAs in other studies. In fact, the five candidate lncRNAs were generally lowly expressed across all three cohorts. However, RNA-seq data revealed a higher proportion of expression for these five genes in patients who relapse compared to non-relapsed patients.

Furthermore, guilt-by-association results correlated *lnckb.61426* to *NOTCH3*, a gene overexpressed in primary leukemia cells from high-risk patients [27], *lnckb.32044* and *lnckb.66409* to *CFD*, a complement regulatory gene linked to poor outcome in AML [28], and *lnckb.66409* to *PVR*. The transmembrane glycoprotein encoded by *PVR* mediates cell adhesion, and was recently proposed as an epigenetic regulator for immunotherapy response in Multiple Myeloma [29]. In that study, similar to the UVC analyses of *lnckb.66409* in our cohorts, higher expression of *PVR* was correlated to a worse outcome. These associations reinforce the hypothesis that the identified lncRNAs may play critical roles in leukemogenesis or disease progression. However, the lack of functional information about these lncRNAs makes it challenging to determine whether they act as drivers of pediatric B-ALL or merely serve as biomarkers, highlighting the need for further studies.

One of the primary constraints of the study is the limited sample size of our cohorts, which included only a small number of relapse cases, deriving in a slight overfitting of the model, and did not allow a deeper study within different genetic subtypes. Nonetheless, the analysis of the TARGET cohort, with its larger sample size and proportionally higher number of relapse cases, lends credibility to our findings regarding the identification of high-risk patients. However, surviBALL’s predictive value should be interpreted with caution and ideally confirmed in larger, independent cohorts.

## Conclusions

In conclusion, surviBALL shows promise for enhancing current risk stratification in pediatric B-ALL, particularly by identifying patients at high risk of relapse at diagnosis, and supporting more personalized treatment strategies. Interestingly, in the current era of data science, traditional diagnostic protocols for pediatric B-ALL risk stratification often rely on multiple tests, which could potentially be streamlined through RNA-seq. This technology enables the simultaneous analysis of molecular subtypes, small variants, fusion genes, expression profiles, and karyotypes in a cost-effective manner, making its application feasible in near real-time clinical settings [12, 30]. As a hypothesis-generating proof of concept, surviBALL could be integrated into existing RNA-seq pipelines to improve patient characterization and risk assessment at diagnosis. Further validation in independent cohorts is needed to confirm its predictive capacity and clinical utility.

## Supplementary Information

Supplementary Material 1.

Supplementary Material 2.

Supplementary Material 3.

## Data Availability

RNA-sequencing data is available at the Sequence Read Archive (SRA) under accession numbers PRJNA850185, PRJNA1237231, and PRJNA1355161. Code used for all the analyses is available at https://github.com/uillar/surviBALL.

## Abbreviations

ALL: Acute Lymphoblastic Leukemia
ALASSO: Adaptive least absolute shrinkage and selection operator
AUC: Area under the curve
B-ALL: B-cell Acute Lymphoblastic Leukemia
COG: Children’s Oncology Group
DFCI: Dana-Farber Cancer Institute
EFS: Event-free survival
HR: Hazard-ratio
ICI: Integrated calibration index
IPA: Index of prediction accuracy
lncRNA: Long non-coding RNA
lncRNAKB: lncRNA Knowledgebase
miscRNA: Miscellaneous RNA
MRD: Minimal residual disease
ncRNAs: Non-coding RNAs
PC: Protein-coding
PETHEMA: Programa Español de Tratamientos en Hematología
RNA-seq: RNA sequencing
ROC: Receiver operating characteristic
SEHOP: Sociedad Española de Hematología y Oncología Pediátricas
TARGET: Therapeutically applicable research to generate effective treatments
UVC: Univariate Cox proportional hazards
