# Supplementary material for: surviBALL: exploring lncRNA expression at diagnosis for 5-year EFS risk stratification in pediatric B-ALL—a proof of concept
Source: Mol Cell Pediatr. 2025 Nov 10;12:19. doi: 10.1186/s40348-025-00210-3 (PMC12597855; doi:10.1186/s40348-025-00210-3)
Supplement: Supplementary file 3 — Supplementary Material 3. [file 40348_2025_210_MOESM3_ESM.pdf]

Article title
surviBALL: Exploring lncRNA Expression at Diagnosis for 5-Year EFS Risk Stratification in Pediatric B-ALL—A Proof of Concept
Journal name
Molecular and Cellular Pediatrics
Authors
Unai Illarregi, Nerea Bilbao-Aldaiturriaga, Angela Gutierrez-Camino, Ivan Martinez de Estibariz, Javier Arzuaga-Mendez, Mireia Camos, Manuel Ramirez-Orellana, Itziar Astigarraga, Chantal Richer, Daniel Sinnett, Idoia Martin-Guerrero, Elixabet Lopez-Lopez.
Correspondence: Elixabet Lopez-Lopez, Department of Biochemistry and Molecular Biology, Faculty of Science and Technology, University of the Basque Country (UPV/EHU), Barrio Sarriena s/n, 48940 Leioa, Basque Country, Spain; email: elixabet.lopez@ehu.eus

Additional File 3. Supplemental figures for *surviBALL: Exploring lncRNA Expression at Diagnosis for 5-Year EFS Risk Stratification in Pediatric B-ALL—A Proof of Concept.*

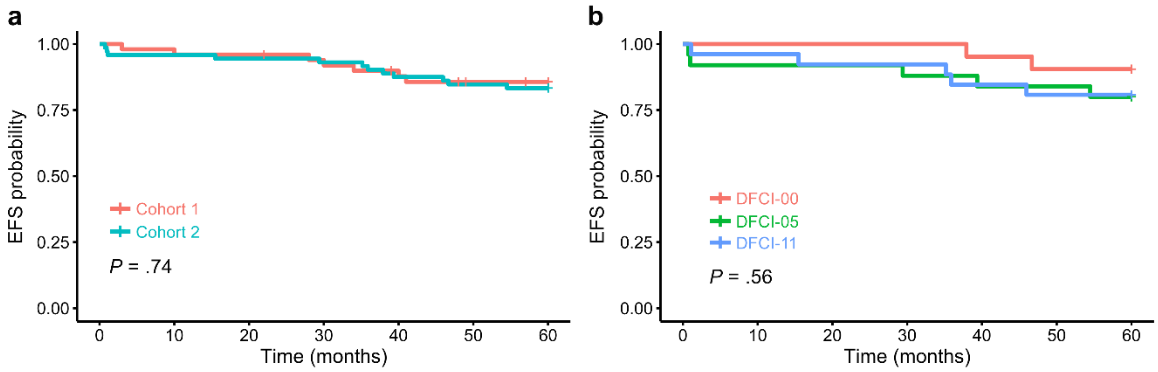

Additional File 3.a a. Kaplan-Meier survival curve for 5-year EFS comparing both populations of study. **b.** Kaplan-Meier survival curve for 5-year EFS comparing different treatment protocols in C2.

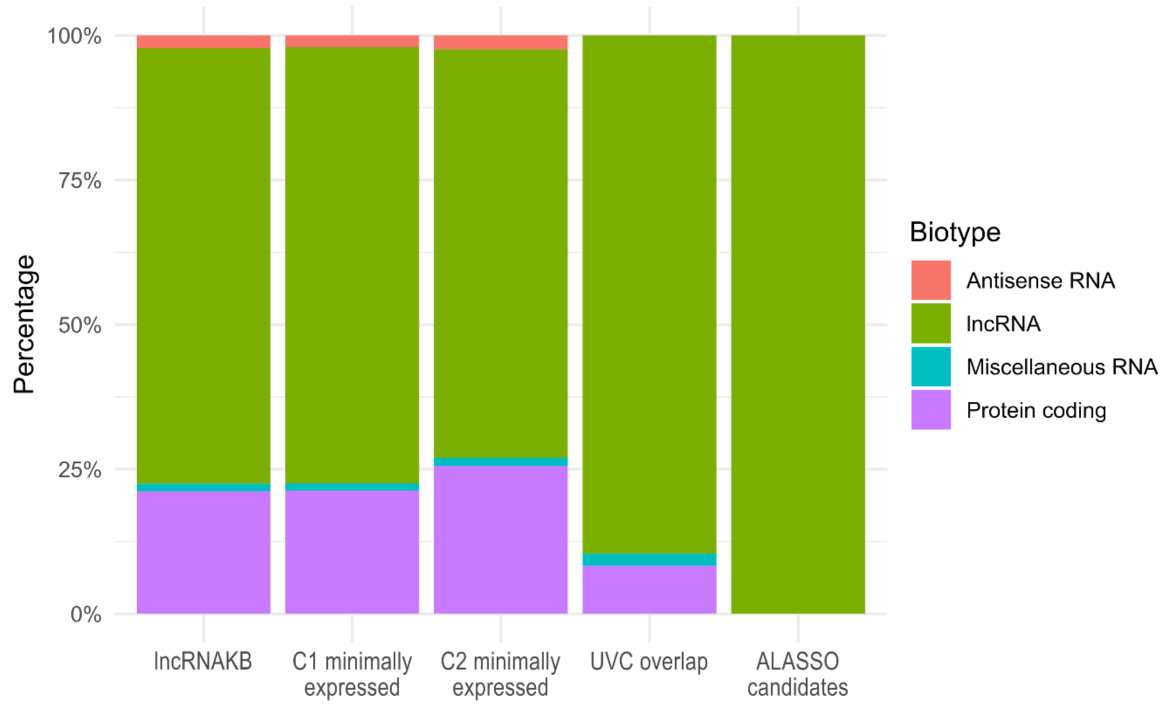

Additional File 3.b Gene biotype proportion comparison at different steps of the study.

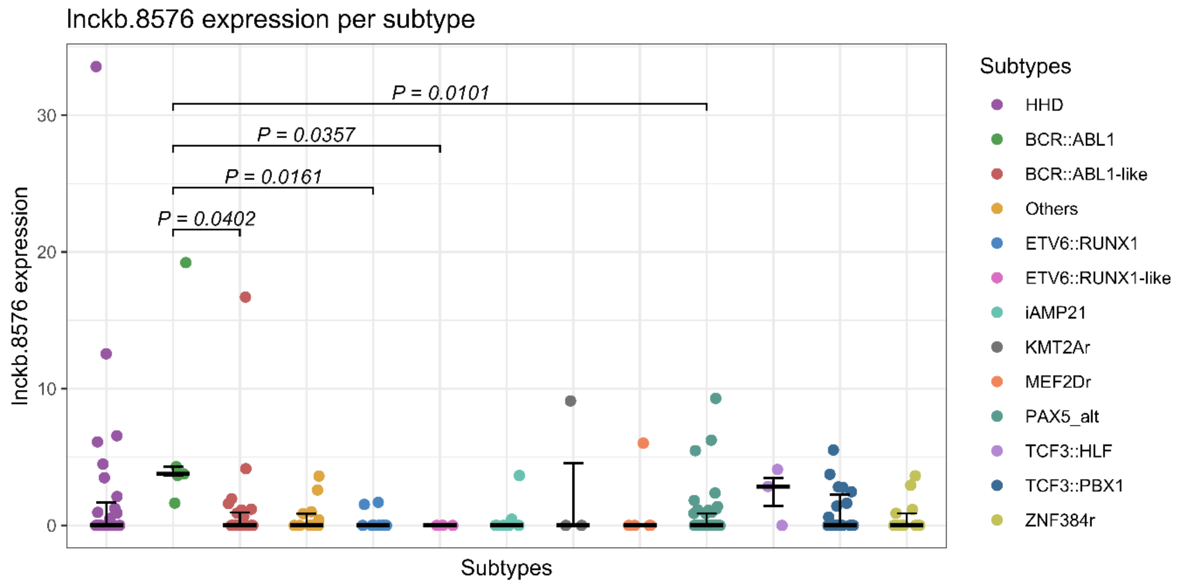

Additional File 3.c Boxplot showing gene expression comparison across subtypes in TARGET cohort for *lnckb.8576*.

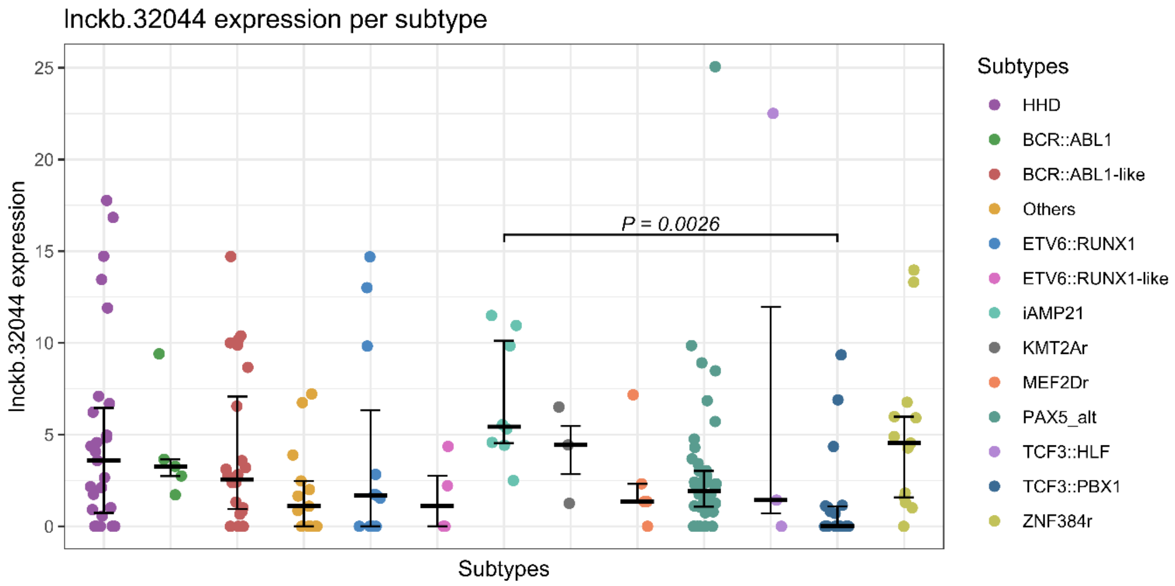

Additional File 3.d Boxplot showing gene expression comparison across subtypes in TARGET cohort for *lnckb.32044*.
